# Politeness when native Japanese speakers and Uzbek Japanese learners give warnings about prohibited acts: a cross-sectional study

**DOI:** 10.12688/f1000research.130926.1

**Published:** 2023-10-27

**Authors:** Takashi Ninomiya, Masaki Ono, Munojot Umarova

**Affiliations:** 1 Faculty of Oriental Studies, Al-Farabi Kazakh National University,, Almaty, Kazakhstan; 2 Faculty of Humanities and Social Sciences, University of Tsukuba, Tsukuba, Japan; 3 Inovation Center, Uzbekistan State World Languages University, Tashkent,, Uzbekistan

**Keywords:** Warning of prohibitions, representative, directive, politeness strategies, native Japanese speakers, Uzbek Japanese learners

## Abstract

**Background:**

Based on singns prohibitiing certain action, people warn othersusing imperative forms of the verb or give a soft warning

Based on signs prohibiting certain actions, people warn others using imperative forms of the verb or give a soft warning using politeness strategies. This study investigates actual situations in which native Japanese speakers and Uzbek learners of Japanese give warnings about prohibitions.

**Methods:**

This study compared warnings given by native Japanese speakers and Uzbek Japanese learners regarding prohibited acts. After clarifying the difference between prohibition and warning of prohibitions, we proposed a classification scheme for the latter speech act in terms of politeness. Data were elicited using a discourse completion task.

**Results:**

The results showed that the Japanese speakers tended to mitigate warnings by implementing two politeness strategies: expression in hedged forms and positioning the warning as a rule. The Japanese speakers used expressions such as “It is prohibited” to indicate that they warned as a rule. Such expressions may cause the hearer to feel that the speaker is an overbearing person who wields authority. To avoid this risk, the Japanese speakers used hedges, such as “Looks like it’s prohibited,” or “It says it’s prohibited,” to make it clear that the right to forbid is not with them but with the public authority. In contrast, the Uzbek learners of Japanese tended to implement their politeness strategy as an apology to compensate for the explicit warning. They tended to apologize in situations where the Japanese speakers would not.

**Conclusions:**

The present study presented a framework for categorizing warning of prohibitions. This framework can be applied to languages other than Japanese, Uzbek, and Japanese by Uzbek learners.

## Introduction

This study is interested in the relationship between signs indicating prohibitions and linguistic expressions in countries around the world, especially in Asian countries. We are interested not only in what kind of linguistic expressions are used in signs indicating prohibitions, but also in how a speaker of a language would warn someone who is committing a prohibited act after seeing a prohibition sign. For instance, they can order a person smoking in a non-smoking area not to smoke, request the person not to smoke, or inform the person of the fact that smoking is prohibited. We are interested in warning strategies in Japanese and other Asian languages. Interests include not only Japanese as a first language, but also Japanese as a second language. The purpose of this study is to clarify pragmatic features when native speakers of Japanese and Uzbek learners of Japanese give warnings about prohibited acts. Japanese and Uzbek, both of which are Asian languages, are agglutinative and have a subject-object-verb structure and similar usage of auxiliary verbs: *e.g.*, both *-te miru* in Japanese and *-b koʻrmoq* in Uzbek mean “to try to *verb*” using verbs derived from the word “to see” ( Yamazaki 2017). For this reason, one expects that Uzbeks will readily learn Japanese grammar. However, even if learners are familiar with Japanese grammar and can produce grammatically correct sentences, they may not be able to successfully implement Japanese speech acts that include warning about prohibitions.

There are two reasons for focusing on Uzbek learners of Japanese. First, as Japanese language education has flourished in Uzbekistan in recent years ( Iwasaki & Umarova 2019: 231), we expect to develop research on Uzbek learners of Japanese. In order to avoid communicative misunderstandings between Uzbek Japanese learners and native Japanese speakers, it is necessary to clarify the learners’ pragmatic characteristics. Second, our 2021 survey ^1^ examined warning of prohibitions by native speakers of Japanese, as well as by Chinese, Korean, and Uzbek learners of Japanese, and failed to characterize Uzbek learners. To elucidate these characteristics, this study raises the following research questions: Which politeness strategies do native Japanese speakers and Uzbek Japanese learners use more frequently when warning of prohibitions?; what pragmatic features other than the politeness strategies do the two language groups display?

### Previous studies

*Studies of speech acts and politeness*

Warning of prohibitions is a speech act taken to avoid possible bad consequences after someone (the “hearer” or “addressee”) is observed to perform a prohibited act. Searle (1976) proposed five basic kinds of illocutionary acts: representative, directive, commissive, expressive, and declaration. Of the five illocutionary acts, Erler (2020: 38) classified the speech act of prohibition as directive. Bach and Harnish (1979: 49) defined prohibition as follows:

“The speaker believes that the utterance prohibits the hearer from doing something because of the authority the speaker has over the hearer; the speaker expresses the intention that the hearer should not do what is prohibited because the speaker says so.”

For example, a guard in a museum can prohibit a visitor from taking photos by saying “Photography is prohibited.” Even though the visitor’s friend says it, they cannot prohibit the act. Since the friend has no authority, they give a warning, not utter a prohibition.

Bataineh and Aljamal (2014: 88) explained warning as follows: “warning refers to the different strategies used for getting the attention of the addressee and alerting him/her to a specific danger or bad consequences.” By saying “Photography is prohibited,” the friend tries to prevent a bad consequence, such as the museum guard scolding the other party. The same expression can either be a prohibition or a warning, depending on whether the speaker has authority or not. According to Searle (1976), warning can function not only as directive (1a), but also as representative (1b). Searle (1976: 22) maintained that warning “may be either telling you that something is the case (with relevance to what is or is not in your interest) or telling you to do something about it (because it is or is not in your interest).”

(1) a. I warn you to stay away from my wife! (directive)

   b. I warn you that the bull is about to charge. (representative)

Warning of prohibitions is a speech act that can cause the hearer to lose face. Although a speaker can baldly warn the hearer, they can also implement politeness strategies by saying they feel sorry for doing the face-threatening act (FTA). Brown and Levinson (1987) identified 14 positive and 10 negative politeness strategies. For example, a speaker can use questions or hedges or adopt the word “sorry” as a politeness strategy in warning situations.

*Studies of prohibition and warning of prohibitions*

Hashimoto *et al.* (1992) investigated warning of prohibitions in eight languages including Japanese and proposed 10 categories of this particular speech act. In the “No photography” situation, the Japanese used the category “Value judgment about a rule” (e.g., Koko de satsuee-shite wa ikemasen “It’s wrong to take photos here”) most frequently. The study created a category of warning of prohibitions and conducted a quantitative analysis. The study then formulated a classification system focusing on social parameters such as status, but did not analyze and discuss how warnings are given in terms of politeness. Previous studies on prohibition have often covered linguistic landscapes: e.g., signboards in public places in Japanese and Korean ( Kim 2011), and signs on university campuses in China and Japan ( Wang 2017). Kishie (2008) analyzed prohibition expressions in the “No dumping” situation in the Tokushima dialect. He established the following five categories: Direct Prohibition I (e.g., Suteruna “Don’t throw out the trash!”), Direct Prohibition II (e.g., Sutetara dame “It’s wrong to throw out the trash”), Indirect Prohibition (e.g., Suterarenaiyo “It’s not possible to throw out the trash”), Request I (e.g., Sutenaide “Don’t throw out the trash!”), Request II (e.g., Motte kaeriyo “Take your trash and go home!”), and Reason (e.g., Kyoo wa gomi no hijanaiyo “It’s not garbage day today”). Because his description of some categories was insufficient, the precise forms of the categories are not entirely clear. The definition of categories in Kishie (2008) requires some caution. For instance, concerning Request I, Kishie (2008: 41) gave only the imperative form, stating that it is used when the speaker requests the hearer not to throw garbage away. However, the study of Blum-Kulka *et al.* (1989) set nine levels of request in terms of indirectness. If Request I corresponds only to the imperative form (i.e., mood derivable) and does not include the other categories, it is not appropriate to label the category with the name Request. One should refer to it as the imperative. Also, despite the formal difference between Direct Prohibition I’s Verb-na (prohibited form) and Request I’s Verb-naide (negation + te-form), both are pragmatically classified as imperative in Blum-Kulka *et al.* (1989). Regarding Uzbek prohibition, Kobilova (2020) only enumerated prohibition expressions in Uzbek and English and did not discuss them. The study of prohibition in Uzbek did not indicate a category of prohibition. 

## Methods

### Participants

Since the members of the research project of pragmatics ^2^ are university faculty members, we asked university students and university graduates, for whom data collection is easier, to cooperate in the survey. Survey participants of this study fall into three groups: 36 native speakers of Japanese (Jap.), 36 Uzbek speakers who were learning or had learnt Japanese (UJap.), and 36 native speakers of Uzbek (Uzb.). We focused on Jap. and UJap. surveys and also analyzed the Uzb. data as a reference. With regard to the Jap. group, faculty members from Japanese universities (Ono and Ju) sent a questionnaire in Japanese to Japanese students and colleagues at their own universities, as well as to Japanese faculties and students at universities with whom they knew. Umarova and Turdiyeva, who teach at a university in Uzbekistan, collected the data on UJap. and Uzb. The UJap. data were obtained from Uzbeks who were studying or had studied Japanese at Uzbek or Japanese universities. The Uzb. data were obtained from Uzbek students at Umarova and Turdiyeva’s university, from their Uzbek colleagues, and from Uzbek faculty members and students with whom were acquainted. The UJap. participants’ Japanese language level was basically intermediate ( *i.e.*, level between N3 and N2 of Japanese language proficiency) ^3^. The Uzb. data were collected and analyzed to investigate the possibility of Uzbek interference with the UJap. data.

### Data collection method

The current study collected the data by means of a discourse completion task (DCT). Members of the above research project created scenarios for the DCT with university students and graduates as respondents. The project members assumed that the students would visit a museum and a hospital and would use warnings based on prohibited signs. Situations were set up in which a student sees two prohibited signs in a museum and two prohibited signs in a hospital. In order to investigate the social relationship between the speaker and the hearer, the members created eight scenarios, focusing on the social parameters of social distance (SD) ^4^ and age as shown in Table 1 (see extended data for English translation of the scenarios).

**Table 1.** Discource completion task (DCT) scenarios.

		Situations	Social Distance (SD)	Age
Situation 1	i	No photography	-SD	x=y
	ii	No entry	-SD	x=y
Situation 2	i	No photography	-SD	x<y
	ii	No entry	-SD	x<y
Situation 3	i	No food or drink	+SD	x=y
	ii	No mobile phone	+SD	x=y
Situation 4	i	No food or drink	+SD	x<y
	ii	No mobile phone	+SD	x<y

See Situation 1-(i) and Situation 2-(i). Although the situations are the same, the social parameters with the hearers are different. In Situation.1, the speaker warns a friend who is around the same age as the speaker (x=y). Also, in Situation. 2, the speaker warns a professor who is older than the speaker (x<y). In Sit.3 and 4, the speaker warns a stranger (+SD). The respondents had to consider the social distance and age between himself/herself and the person who was about to commit the prohibited act. The DCT required the respondents to say something in response to the offender’s action, based on a sign that showed what was prohibited. After creating the scenarios, we created a questionnaire form in Google form (see extended data) and sent the link to the Jap., UJap., and Uzb. groups. The Jap. participants performed the task in Japanese, the Uzb. participants did the task in Uzbek, and the UJap. participants did the task in both Uzbek and Japanese. We gave the participants descriptions of the four situations to elicit warning expressions about a prohibition in oral conversation. At the beginning of the questionnaire, it was stated that only those who were willing to cooperate with the survey should respond and that we would be careful with their personal information. The response data from the Google form was exported to an Excel file (see underlying data). In case of incomprehensible data, we contacted the respondent and asked (the incomprehensible data were marked in yellow on the sheet).

### Data analysis

We created a scheme, based on request and prohibition studies: Blum-Kulka *et al.* (1989), Trosborg (1995), Kishie (2008), and Bella (2012). We presented two head acts and one supportive move. A head act is the minimal unit which can realize a warning of prohibition and is the core of the warning sequence. Supportive move is a unit external to the warning of prohibition, which modifies its impact by mitigating its force. The two head acts consist of the request-type and prohibition-type as presented by Kishie (2008). The request-type has the following eight categories in terms of indirectness: Level 1 imperative “Don’t take photos!”; Level 2 explicit performative “I ask you not to take photos”; Level 3 hedged performative ^5^ “I would like to ask you not to take photos”; Level 4 obligation statement “You should not take photos”; Level 5 want statement “I want you not to take photos”; Level 6 suggestory formula “How about not taking photos?/Let’s not take photos”; Level 7 query preparatory “Could you not take photos?”; Level 8 hint ^6^ “You can take photos in another place.” We called these categories Directive-Warning (DW) because the request-type categories can only be interpreted as directive.

As the second head act, we proposed Representative-Directive-Warning (RDW), which corresponds to Kishie’s (2008) prohibition-type. This type has the following categories: Level 1 explicit infringement-indication “Photography is prohibited”; Level 2 hedged infringement-indication “Looks like photography is prohibited”; Level 3 fact-checking “Isn’t it wrong to take photos?” As indicated in section “Studies of speech acts and politeness,” warning functions as directive and representative acts. The sentence “Photography is prohibited” functions as a directive in that the speaker stops the hearer from being about to take photos, but superficially it seems to be representative. Since there are sentences that have both representative and directive features, we made the RDW-type. In RDW-type expressions, the speaker points out that the hearer’s action is morally or legally problematic. As Blum-Kulka *et al.* (1989) classified explicit and hedged performatives as request strategies, this study classified explicit and hedged infringement-indications in the RDW-type categories. The RDW-type’s fact-checking corresponds to the DW-type’s query preparatory. Observing examples of this type from a different point of view than indirectness, we found that there are characteristic differences between Jap. and UJap. in the form of the predicates. We set up four predicate forms, “Prohibited to Verb”-form, “Not possible to Verb”-form, “Wrong to Verb”-form, and “Right to Verb”-form (Verb is hereafter abbreviated as V), and found a difference in their utilization between the Jap. and UJap. groups.

Based on Blum-Kulka *et al.* (1989) and Bella (2012), we made the following classification scheme for supportive moves: (i) preparator “I have a favor to ask. (Can you stop calling here?)”; (ii) getting a precommitment “Can I have a word with you? (Looks like drinking isn’t allowed)”; (iii) grounder “There is a sign that says, “Staff only!” (You can’t enter there)”; (iv) alternative “(You can’t enter there) I’ll show you to a restroom”; (v) apology “Sorry. (Photography is prohibited here).” One can place the first and second categories not only in the context of request, but also in the context of warning. The third category grounder is the external mitigation where the speaker gives reasons, explanations, or justifications for his/her warning. The fourth alternative corresponds to Blum-Kulka *et al.*’s (1989) “promise of reward.” In the case of a request, the speaker may give the hearer a reward, but in the case of a warning, the speaker may suggest to the hearer an alternative plan to replace the prohibited act instead of giving a reward. Regarding the fifth category, Bella (2012), who investigated linguistic mitigation in Greek requests, set this category as one of the supportive moves.

Based on the above, this study created two head acts, the DW-type (eight subcategories) and the RDW-type (three subcategories), and one supportive move (five subcategories). The RDW-type was also classified into four categories according to predicate form. In the utterance of a warning of a prohibition, either a Head act of the DW or RDW-type is mandatory, but a supportive move is not necessarily mandatory.

We obtained the following data sets from the participants: The number of Jap. data 288, Uzb. 288, and UJap. 288 (total 864). We labeled all the utterances pertaining to warning of prohibitions as follows.

(2) Byooin desukara, koko de tabe tari, non dari wa ikemasen.

  Grounder     Explicit infringement-indication (Wrong to Verb)

  This is a hospital, so it’s wrong to eat or drink here.

The example (2) above is from Situation 3-i “No food or drink” in Uzbek Japanese. All labeling data is placed under the underlying data. In the example, one explicit infringement-indication for the RDW-type, and one grounder for the supportive moves. In the RDW-type, one “Wrong to V”-form was counted. We counted the number of the subcategories of the DW-type, RDW-type, and supportive moves for each of the eight scenarios. We labeled the data that indicated no warning as “Don’t do FTA,” and the number of them was also shown. We also calculated the percentages of the subcategories of the DW-type and RDW-type. The number of each subcategory was divided by the total number of head acts. To calculate the utilization rate of the four predicate forms of the RDW-type, the number of each predicate form was divided by the total number of RDW-types. To calculate the utilization of the three subcategories of supportive moves, the number of each subcategory was divided by the total number of supportive moves. In addition, we analyzed each social parameter. Since there were many apologies among the supportive moves, we counted the number of apology in each social parameter ( *i.e.*, social distance and age), and divided the number of apology by the total number of apology for each social parameter. To obtain the utilization rate of “Don’t do FTA” for each social parameter, we counted the number of “Don’t do FTA” for each social parameter, the number of each social parameter was divided by the total number of “Don’t do FTA.” To obtain the utilization rate of the DW-type, RDW-type, supportive moves, and “Don’t do FTA,” the number of those four semantic formulae was divided by the total number of semantic formulae. Calculations were made using Microsoft Excel (see extended data).

## Results

Table 2 indicates the DW-type categories and examples found in the Jap. and UJap. data. Contrary to our expectations, we did not find any examples of categories other than the imperative, want statement, and hint. Also, we did not find any examples of categories other than imperative, obligation statement, suggestory formula and hint in the Uzb. data ^7^. Negative imperative forms that would stop the hearer’s action were classified as imperative. The desiderative form in which the speaker wishes hearer to stop action, were classified as want statements ^8^. Vague warning without a specific form was classified as hint. We can say that these three languages lack diversity in the DW-type.

**Table 2.** Directive-Warning type categories in Japanese and Uzbek Japanese.

Level	Categories	Examples
1	Imperative	Japanese: *Shashin o toranaide kudasai* “Don’t take photos!”; Uzbek Japanese: *Koko de shashin o toranaide* “Don’t take photos here!”
2	Explicit performative	—
3	Hedged performative	—
4	Obligation statement	—
5	Want statement	Japanese: ( *Koko satsuee-kinshi dakara*) *yameyoo* “(It is prohibited to take photos here, so) I want you to stop doing”; Uzbek Japanese: —
6	Suggestory formula	—
7	Query preparatory	—
8	Hint	Japapnese: *Soko toirejanaiyo* “That’s not a toilet”; Uzbek Japanese: *Kochira wa sutaffuyoo mitai desu* “This seems to be for the staff”

Table 3 shows the categories of the RDW type. We gave the auxiliary verb *mitai* “looks like” as an example of hedged infringement-indication in the table. In addition, there are also hedges such as *omoimasu* “I think that” and *tte kaite aru* “It says that” in both Jap. and UJap. verbal phrases. Regarding the category fact-checking, this study shows that Jap. had examples of this but UJap. did not.

**Table 3.** Representative-Directive-Warning type categories in Japanese and Uzbek Japanese.

Level	Categories	Examples
1	Explicit infringement-indication	Japanese: *Koko wa satsuee-kinshi desuyo* “It is prohibited to take photos here”; Uzbek Japanese: *Kochira wa satsuee-kinshi desu* “It is prohibited to take a photo here”
2	Hedged infringement-indication	Japanese: *Shashin-satsuee dame mitaiyo* “Looks like photography is wrong”; Uzbek Japanese *Koko de shashin totte wa ikenai mitaiyo* “Looks like it’s wrong to take photos here”
3	Fact-checking	Japanese: *Soko haiccha damejanai?* “Isn’t it wrong to enter there?”; Uzbek Japanese: —

When the predicate of a warning sentence had any of the four predicates listed in section “Data analysis” and the infrigment was explicitly noted without hedging, the sentence were classified as explicit infrigment-indication. When the predicate of infrigment-indication was hedged, the sentence were classified as hedged infrigment-indication ^9^. For sentences in which the hearer is asked if his or her action is prohibited, the sentence was classified as fact-checking. The sentences in which explicit infrigment-indication is interrogativized were classified into this category.

Figure 1 presents the percentages of each category of the head acts for which at least one example was found. Regarding explicit infringement-indication, which is the most direct in the RDW-type, the UJap. group used it almost twice as much as the native groups. In regards to hedged infringement-indication, which is more indirect than explicit infringement-indication, the Jap. group used it about 1.5 times more than the other groups. The Jap. participants used very few imperative compared to the UJap. and Uzb. participants. Regarding jint, Uzb. used it the most and UJap. used it the least.

**Figure 1.**
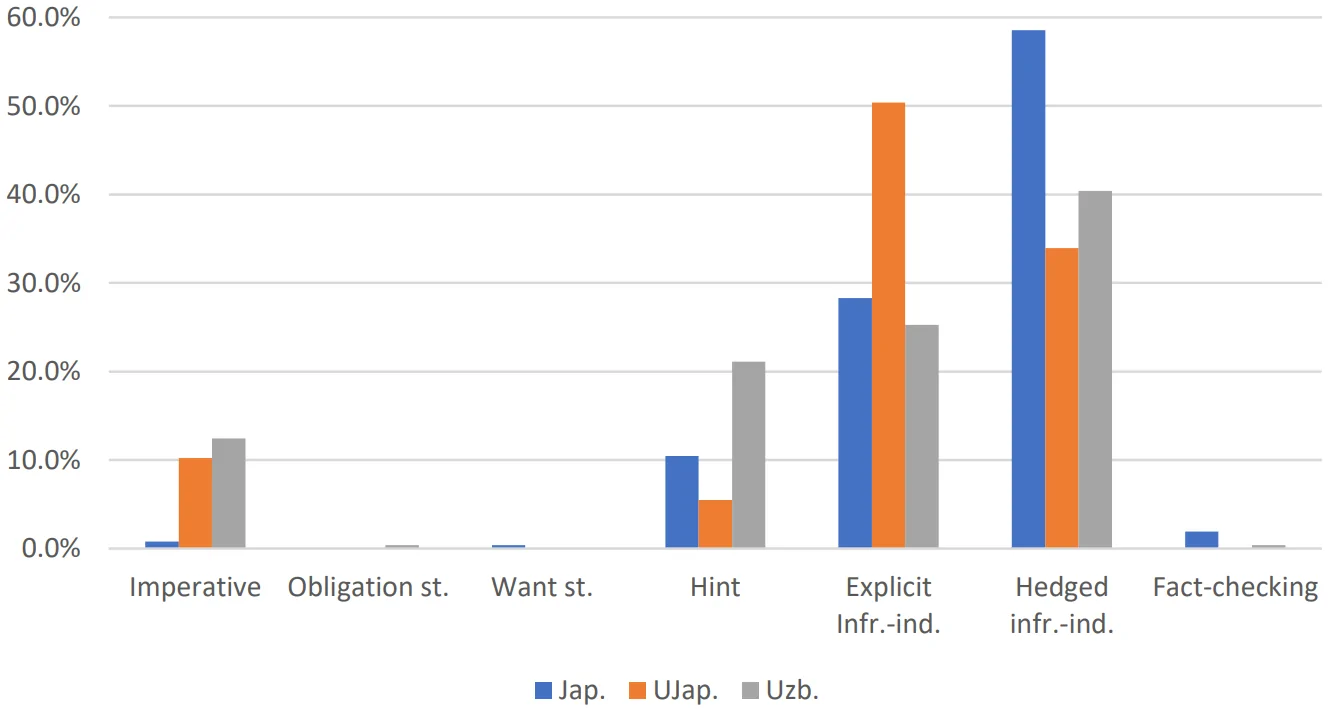
Percentages of head acts (Directive-Warning and Representative-Directive-Warning types)’ categories.

As shown in Table 4, we divided the three RDW-type categories into four in terms of predicate form. The first “Prohibited to Verb”-form means that the hearer’s action is not permitted by the rule. The second “Not possible to Verb”-form has the meaning that the hearer cannot perform the action. Using the third “Wrong to Verb”-form and the fourth “Right not to Verb”-form, the speaker communicates that the hearer’s act is bad and not good based on the speaker’s value judgment ^10^. If the speaker warns by using the second, third and fourth forms without adding an expression like “It’s a rule” or “There’s a no photography sign over there,” the basis for their warning is not due to a rule, but a value judgment. However, if the speaker uses the first form, they can warn the hearer on the basis of the rule, even without reason for the waring. Concerning the third “Wrong to Verb”-form, we set two forms: *dame* and *ikenai.* As described below, we found differences in the utilization of these two forms between the Jap. and UJap. groups.

**Table 4.** Predicate forms of Representative-Directive-Warning type in Japanese and Uzbek Japanese.

Form names	Japanese and Uzbek Japanese forms
Prohibited to Verb	*kinshi da*
Not possible to Verb	*dekinai, kibishii*
Wrong to Verb	*dame, ikenai*
Right not to Verb	*shinai hoo ga ii*

Figure 2 shows the percentages of the four predicate forms in the RDW-type categories. The Jap. group used the “Prohibited to Verb”-form which conveys the fact of prohibition most frequently at about 70%. The Uzb. group used the “Not possible to Verb”-form most frequently at about 50%. The UJap. group used “Wrong to Verb”-form as often as “Prohibited to Verb”-form at about 40%, respectively. When subclassifing the “Wrong to V”-form in the Jap. and UJap. data, in the Jap. group, we found 36 words for *dame* and 8 words for *ikenai*, and in the UJap. group, we found 19 words for *dame* and 58 words for *ikenai.* In other words, for the “Wrong to Verb”-form, the Jap. group used the word *dame* more frequently, while the UJap. group used the word *ikenai* more frequently.

**Figure 2.**
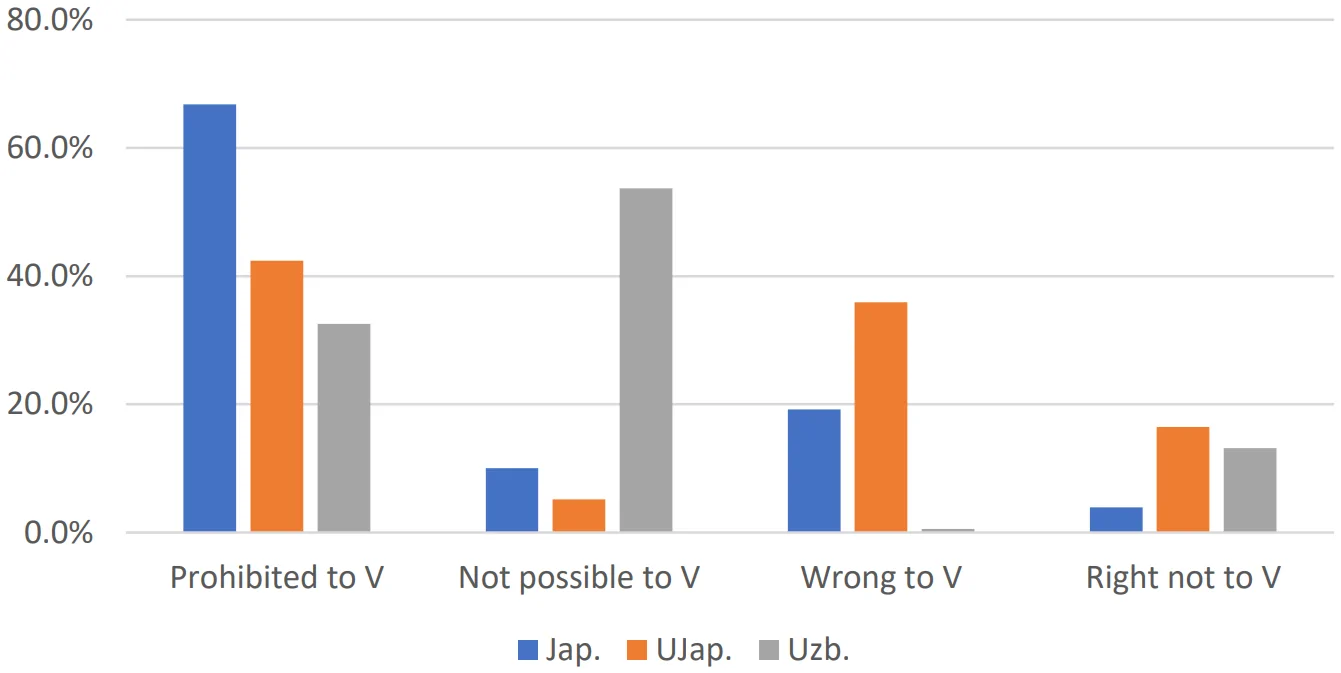
Percentages of Representative-Directive-Warning type’s predicate forms.

As Table 5 shows, Jap. and UJap. did not produce any examples of preparator and getting a pre-commitment in the supportive moves. The Uzb. participants also gave no examples of these categories. The DCT data revealed that apology ^11^ almost always precedes a head act.

**Table 5.** Supportive moves in Japanese and Uzbek Japanese.

Categories	Examples
Preparator	—
Getting a Precommitment	—
Grounder	Jap. *Koko wa kankeesha-no-hito-no-basho dakara (watashitachi wa hairenai tokoro dayo)* “This place is for authorized persons, so (it’s a place where we can’t enter)”; UJap. *Soko wa sutaffu tame-no-toire da, (hairanai hoo ga iiyo).* “That’s a staff bathroom. (It’s better not to go in there)”
Alternative	Jap. *(Koko keetai-denwa tsukaccha dame mitainanode,) denwa wa soto de shita hoo ga ii desuyo* “(As it’s wrong to use your cell phone here,) it’s better to use outside”; UJap. *(Koko wa inshoku kinshinanode,) chotto soto ni itte, nonda hoo ga iinjanai desu ka?* “(Food and drink are prohibited here, so) wouldn’t it be better that you go outside and drink it?”
Apology	Jap. *Sumimasen. Koko inshoku-kinshi desuyo* “Sorry. Eating and drinking is prohibited here”; UJap. *Mooshiwake-arimasen-ga, (inshoku kinshi-sa-rete-imasu-yo.)* “I’m sorry, but (food and drink are prohibited)”

As shown in Figure 3, Apology was the most frequent in the supportive moves. The UJap. group used Apology about 20% more frequently than the native groups.

**Figure 3.**
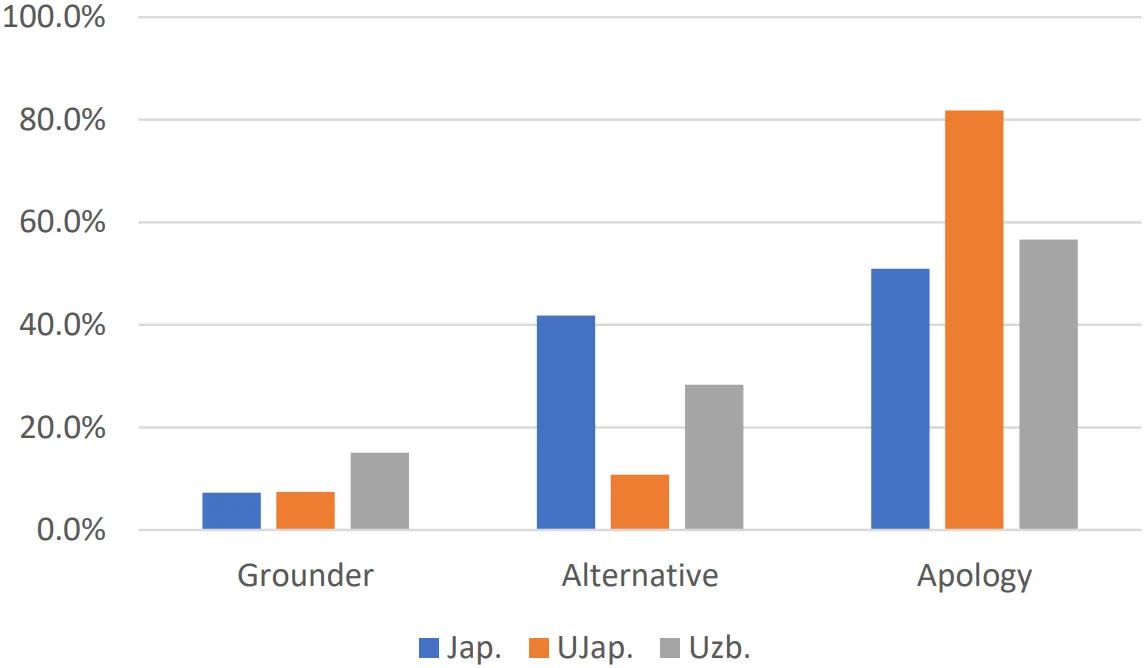
Percentages of supportive moves’ categories.

Figure 4 shows the number of Apologies by social parameters. While Jap. apologized only to +SD, namely unknowns, UJap. apologized to not only +SD but also -SD, whom the speaker knows well. However, for -SD and x=y, the hearers who are close to the speaker, the UJap. group did not apologize as much.

**Figure 4.**
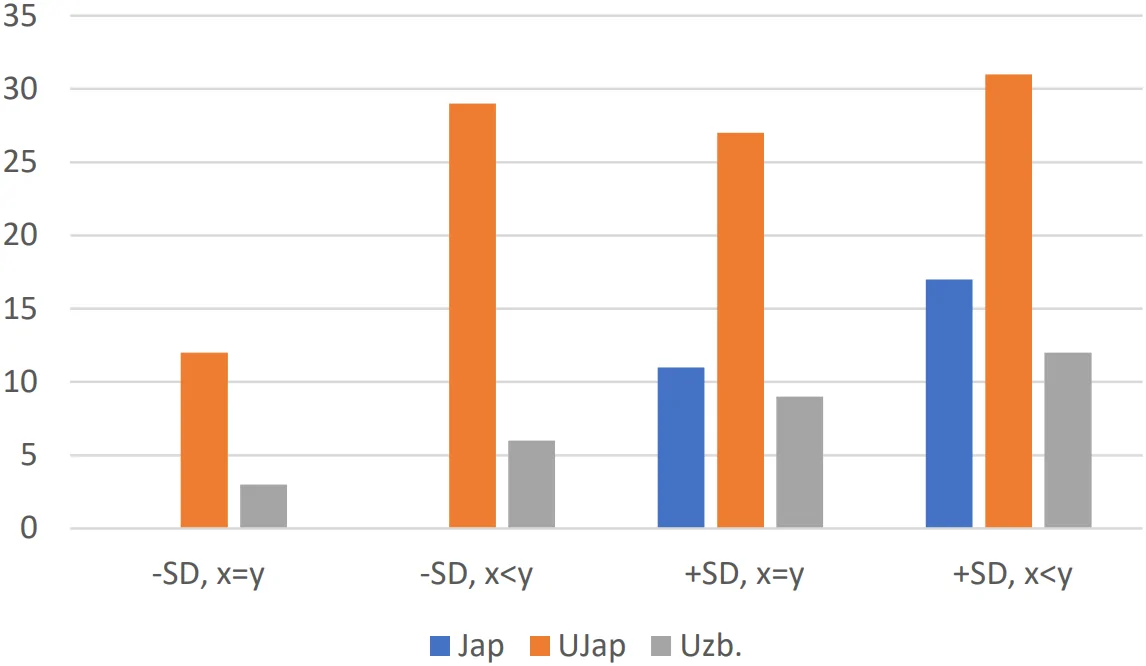
Number of apologies by social parameter.

Participants noted that they do not warn the hearer in some situations. We categorized the case as “Don’t do FTA.” Table 6 indicates the number and percentage of DW, RDW, Supportive moves, and Don’t do FTA. The total number was highest for the UJap. group. The group used Supportive moves more than the other groups, about twice as often. All the groups used more the RDW-type than the DW-type. In particular, Jap. used RDW about eight times more frequently than DW.

**Table 6.** Number and percentage of Directive-Warning, Representative-Directive-Warning, Supportive moves, and Don’t do “face-threatening act (FTA)”.

	Japanese	Uzbek Japanese	Uzbek
Directive-Warning	30 (8.3%)	43 (10.2%)	92 (24.5%)
Representative-Directive-Warning	229 (63.6%)	231 (54.6%)	177 (47.1%)
Supportive moves	55 (15.3%)	121 (28.8%)	53 (14.1%)
Don’t do FTA	46 (12.8%)	28 (6.6%)	54 (14.4%)
Total	360	423	376

Figure 5 indicates that all the groups implemented the “Don’t do FTA” strategy for +SD, namely unknowns. Among the three groups, UJap. had the least number of people who implemented “Don’t do FTA,” In other words, the learners’ group warned about prohibitions most frequently.

**Figure 5.**
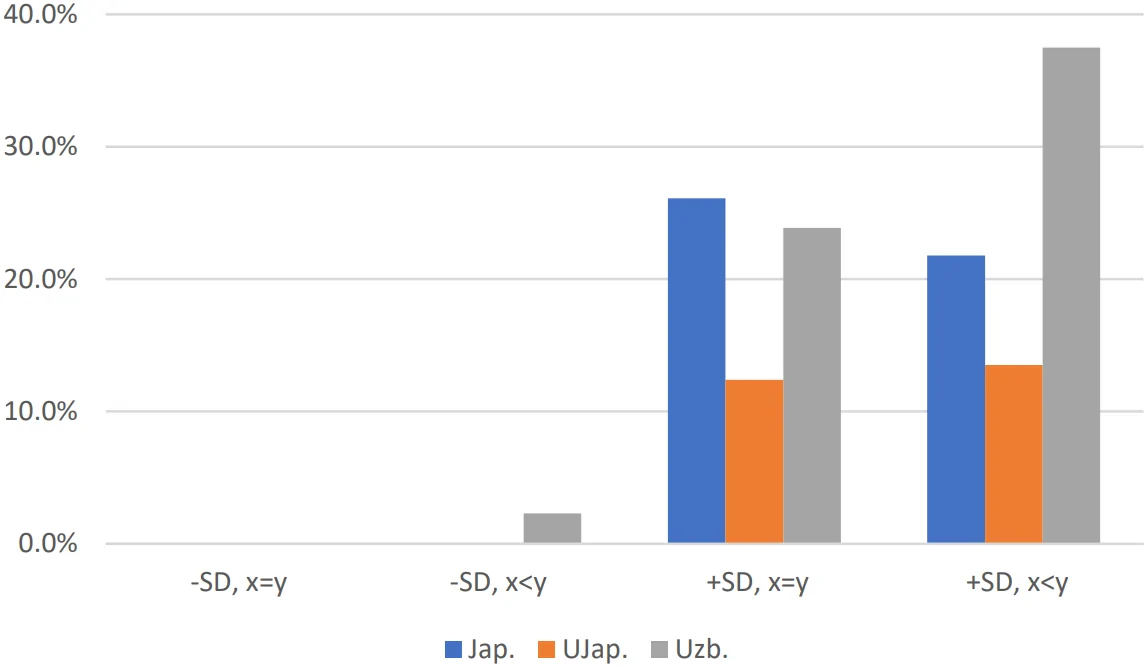
Percentages of Don’t do “face-threatening act (FTA)” by social parameter.

## Discussion

One of the significant findings from this study was that Uzbek learners of Japanese tended to be verbose. Data analysis of Figure 3 and Table 6 revealed that the UJap. group used more supportive moves, especially apology, than the Jap. and Uzb. groups, although the native groups avoided verbose utterances by providing only the necessary information. The verbosity of the learner group would not have been influenced by their native language, but by the characteristics of the learner language. If Uzb. as well as UJap. had a higher total number of semantic formulae, the UJap. verbosity could have been said to be an influence from the native Uzbek language, but this is not likely. Blum-Kulka and Olshtain (1986: 177) asserted that lengthening of speech act patterns and addition of supportive moves in languages spoken by learners, especially advanced learners, are due to the learners’ lack of confidence. They want to get their message across but are not confident in their speaking ability. Cenoz (1995: 5) said that grounder, which provides reasons and explanations to justify the need to make a request, was the most common in the supportive moves that learners used in request. Ito (2002) reported that lengthening of speech act patterns (especially reasons for refusal) occurred in the refusal of Japanese by groups of native Malay speakers. UJap. subjects tended to be at the intermediate level of Japanese language proficiency (N3-N2 levels), but as Blum-Kulka and Olshtain (1986: 177) previously noted, the subjects exhibited “lengthening of speech act patterns” found in advanced level learners.

Unlike the previous studies of request and refusal, this study showed that apology was more common than grounder. In the scenario where the spaker warned about a prohibition, many Uzbek learners of Japanese may have assumed that the speaker does not need to actively explain the reason for the warning because the situation pertaining to the warning is clear and that the speaker expects the hearer to know that one is not allowed to take pictures in certain areas of a museum or use their cell phone in certain areas of a hospital. For this reason, UJap. participants probably implemented the negative politeness strategy of apology to express their feeling of being sorry for having to issue a warning rather than to give reasons for the warning. Also, UJap. participants apologized to both strangers and those who they knew, while Jap. participants apologized only to strangers. The more extensive use of apology in UJap. than in Jap. means that Uzbek learners of Japanese may apologize more readily than Japanese.

An analysis of the head acts, the core of warning of prohibitions, revelaed that Jap. warning was indirect and that it was a rule-based warning, while UJap. warning was direct and that it was based on the speaker’s value judgment. See the RDW-type’s categories in Figure 1. The results showed that the Jap. group used indirect hedged infringement-indication more frequently than the UJap. and Uzb. groups and that the UJap. group used direct explicit infringement-indication more frequently than the Jap. and Uzb. groups. The DW-type’s data indicated slightly that Jap. was indirect and UJap. was direct. While the Jap. group did not use the highly direct Imperative at all, the UJap. group used it only slightly, as did the Uzb. group. Also, Jap. used almost twice as many hint with the highest indirectness than UJap., although the overall number was small. The Jap. group tried to get the hearer to stop the prohibited act by moderately communicating the fact that the hearer’s action was not permitted. In contrarst, the UJap. group tried to get the hearer to stop the action by baldly communicating that based on their opinion, the hearer’s action was wrong. Generally, learners of a language have been found to be more direct than native speakers in some studies ( Cenoz 1995: 5). For instance, Fukushima (1990) pointed out that offer and request by Japanese learners of English were more direct than the forms used by native English speakers. The Jap. indirectness in this study was expressed by the negative politeness strategy of hedge. Many UJap. participants probably did not use hedged forms, because hedged forms in Japanese are difficult for the learners or because the learners do not know that the Japanese use the hedged forms as a politeness strategy.

As Figure 2 shows, the Jap. group used the “Prohibited to V”-form most frequently to convey the fact that the hearer’s action was not permitted by a rule, while in addition to that form, the UJap. group also frequently used the “Wrong to V”-form to convey that the hearer’s rule violation was wrong. Many Jap. participants warned the hearer while indicating the prohibition sign at the same time. This transferred the responsibility for warning to the museum or hospital. In contrast, about half of the UJap. participants themselves took on the responsibility that originally belonged to those institutions and conveyed the opinion that hearer’s action was wrong. The reason the UJap. participants frequently used the “Wrong to V”-form was not the influence of their native language, but the non-native language, because the Uzb. group used the “Not Possible to V”-form most frequently, but rarely used the “Wrong to V”-form. The UJap. group may have frequently used the “Wrong to V”-form due to the influence of a Japanese textbook. Of the “Wrong to V”-forms, Jap. used the word *dame* frequently. However, UJap. used the word *ikenai*, which appears early in Japanese language learning; the word *ikenai* appears in Lesson 15 of a major Japanese language textbook *Minna no nihongo elementary I* ( Three A Network 2012: 126). The UJap. group may have wanted to give a warning by using the “Prohibited to V”-form but since the expression was difficult, the group may have been forced to use the more user-friendly word *ikenai.* All the language groups used the RDW-type more frequently than the DW-type (see Table 6). It is unclear whether the frequent use of the former type is due to the influence of a specific language, such as Japanese, Uzbek, or Uzbek-Japanese, or not. If the language itself is unrelated to this phenomenon, then some individuals simply may not want to use the DW-type as a warning.

We found that the three language groups did not warn strangers (see Figure 5). When warning violators of the prohibited act, all the groups implemented the “Don’t do FTA” strategy for unknowns. Participants assumed that if they warned an unknown, this could result in a backlash. A further analysis of the figure showed that the UJap. group was more likely not to implement “Don’t do FTA” for an unknown than the Jap. and Uzb. groups. This feature indicates the possibility that Uzbek learners of Japanese may inadvertently warn in situations where native Japanese and Uzbek speakers do not.

Although participants of the three language groups could show indirectness in the DW-type as well as in the RDW-type, they did not use any indirect strategies of the DW-type except for hint. While request categories and warning’s DW-type categories were the same, the frequency of use were different. Harting (2008: 125) showed that in the request situations, more than 50% of the Japanese participants used the highly indirect query preparatory and about 20% of them used the moderately indirect want statement. However, in the warning situations, none of the groups used such indirect DW strategies. The participants may have avoided using such indirect strategies because the strategies were too euphemistic or because directive speech acts were not appropriate for the warning of prohibition scenarios.

The three language groups did not use preparator and getting a precommitment in the supportive moves. In the warning of prohibition scenarios, although the speaker does not have the authority to prohibit, they can serve as a spokesperson for the authority. Because of this role, the participants may have thought that there was no need to use a preparator to inform the hearer that they is to anticipate the warning, nor does speaker need to try to secure a pre-commitment before the warning with getting a pre-commitment. The participants in all the language groups would not have used such circuitous strategies because the speaker does not need to adopt a humble attitude toward the hearer in warning situations.

When compensating for warning, the Jap. group implemented the two politeness strategies of positioning the warning as a rule and of using hedged forms. Brown and Levinson (1987: 206) pointed out that in the strategy “State the FTA as a general rule,” one way of communicating that the speaker does not want to intrude but is merely forced by circumstances is to state the FTA as an instance of rule, regulation, or obligation. With the “Prohibited to V”-form, many Jap. participants did not want to warn, but may have wanted to imply that they had to warn due to the museum’s rule. Although the use of the “Prohibited to V”-form has the advantage of allowing the speaker to transfer the responsibility for the warning to an authority, there is a danger that the expression alone may lead the hearer to feel that the speaker is a high-pressure person who use of authority. To avoid this risk, the Jap. group also frequently implemented the second politeness strategy of hedge. As one of several hedge forms, Brown and Levinson (1987: 152) cited the hedge that may be used to distance the speaker from a command by indicating that the speaker’ speech act is attributed to a third-party command. For example, by saying, *Satsuee-kinshi **tte kaiteruyo*** “It says no photography,” the speaker can explicitly convey that a third-party, the museum, forbids the action. Also, the statement *Soko, tachiiri-kinshi **mitai**dayo* “There, looks like it’s off-limits,” indicates that the hearer’s action is an assumption and uncertainty on the part of the speaker. The speaker may be mitigating his/her accusation by the hedged form *mitai.* Since the UJap. group did not use those politeness strategies as much as the Japanese group, the UJap. participants appear to have issued blunt warnings. However, they implemented the politeness strategy of apology, feeling sorry for doing the FTA. Brown and Levinson (1987: 187, 189) suggested that by apologizing, the speaker communicates that they does not wish the hearer to lose face, thereby partially compensating for the infringement. Many UJap. participants may have believed that the Japanese typically use the apology as a politeness strategy in any situations, or may have thought that an apology was a way of saving the hearer’s face in a situation that could create the tension associated with a warning. The fact that the UJap. participants apologized more frequently than the Jap. participants indicates the possibility that Uzbek learners might apologize unnecessarily even in situations where the Japanese do not apologize. Japanese language teachers should tell the learners that there is no need to apologize before giving a warning.

## Conclusions

Referring to prohibition and request studies, this study created a classification scheme for warning about prohibitions and compared the speech act used by native Japanese speakers and Uzbek Japanese learners. The Japanese participants frequently used hedged infringement-indication in the RDW-type. They implemented the politeness strategy of hedge to convey the content of a warning in a moderate manner. Also, by using the “Prohibited to V”-form, they implemented the politeness strategy in which one states the warning as a rule. Simply using the “Prohibited to V”-form runs the risk that the hearer feels that the speaker is a high-pressure person who wields authority. To avoid this risk, the Japanese speaker used the hedge’s politeness strategy ( *e.g.*, “It says it’s prohibited”) to make it clear that the right to prohibit rests with the public authority, not with themselves. On the other hand, the Uzbek learners of Japanese tended to apologize. Because of frequently using explicit infringement-indication, it seems as if they gave a blunt warning. To compensate for doing the FTA, the learners implemented the politeness strategy of Apology. However, the learners’ overuse of Apology means that they may apologize excessively in situations where the Japanese do not apologize.

We found the two pragmatic features other than the politeness strategies. First, native speakers of Japanese, Uzbek learners of Japanese, and native speakers of Uzbek implemented the “Don’t do FTA” strategy only for unknowns; that is, none of the language groups warned complete strangers. However, as shown in Figure 5, more than half of the participants of all three groups did not respond this way. Each of the DCT’s questionnaire statements did not ask whether the warning was given in the first place. In the DCT, if we ask not only “What do you say to the hearer to warn him/her?” but also “Do you warn the hearer in this situation?,” the majority of the participants may not warn unknowns. Regarding the second pragmatic feature, all the language groups used the RDW-type more than the DW-type. Although the participants could have chosen conventional indirect warning strategies among the DW-type, they did not use them at all. It is not clear whether speakers consider indirect expressions to be too euphemistic in the context of warning of prohibitions, or not. A future survey will allow us to obtain opinions on the use of indirect expressions in the DW-type.

The present study developed a classification scheme for warning about prohibitions, based on the Japanese and Uzbek Japanese data, and referring to the Uzbek data. In the future, we will use this framework to analyze this speech act of learners of Japanese other than Uzbeks and native speakers of Asian languages other than Japanese. By examining data from a large number of languages, we can develop a more general classification scheme, and will clarify why the RDW-type was used more frequently than the DW-type and the reason for the non-use of indirect strategies in the DW-type, as these issues were not fully addressed and clarified in this study. In response to the need to expand the study of Uzbek Japanese, we conducted a pragmatic study focusing on Uzbek learners of Japanese as well as native Japanese speakers. In the past, opportunities for Uzbeks to visit Japan were rare. They studied Japanese to learn about Japanese culture and to improve their Japanese language skills, including grammar, reading and listening comprehension, in preparation for the Japanese Language Proficiency Test. Pragmatic competence as an output may not have been much needed. However, in 2019, Japanese policy made it possible for Japanese companies to accept Uzbeks as technical interns, and many Uzbeks are now coming to Japan. In the future, it will be required to investigate the actual situation of Japanese language use by Uzbek learners from the viewpoint of pragmatics, including warning of prohibitions, and to apply the results to their Japanese language learning.

## Ethics and consent information

Retrospective ethical approval for this study on the 01/05/2023.

All participants provided implicit consent by completing the online questionnaire.

## Data Availability

Figshare: All data of Politeness when native Japanese speakers and Uzbek Japanese learners give warnings about prohibited acts, https://doi.org/10.6084/m9.figshare.24179064.v1 ( Ninomiya 2023). This paper contains the following underlying data:
- Raw data.xlsx - Labelling data: Japanese labelling.docx, Uzbek Japanese labelling.docx, Uzbek labelling.docx This paper contains the following extended data:
- Original scinario questionnaires: Japanese questionnaire.pdf, Uzbek Japanese questionnaire.docx, Uzbek questionnaire.pdf - English translation of questionnaires.docx - Data analysis.xlsx

## Footnotes

^1^
The results of this research were presented at the Symposium on Japanese Language Education at the University of Tsukuba on February 13, 2021: Ninomiya T., Li G., Lina A., Ju Hy., Gao, Y., Umarova M., Turdiyeva X, Li Ts. X., and Ono M. (2021) **“Nihongo bogo washa nihongo gakushuusha ni yoru kinshi irai hyoogen no hyooka: Poraitonesu no kanten kara”** [Evaluation of prohibitive and requestive expressions by native Japanese speakers and learners of Japanese: From the viewpoit of Politeness]. In: Shinpojiumu mirai shikoo no nihongo kyooiku 2.0. [Future-oriented Japanese language education 2.0].

^2^
The presenters in footnote 1 are members of the research group.

^3^
Of the 36 respondents, 27 indicated their level of JLPT: 0 were at N5, 4 at N4, 9 at N3, 12 at N2, and 2 at N1. The majority of the UJap. respondents were N2 and N3, which correspond to intermediate level, while N5 and N4, which correspond to beginner level, and N1, which corresponds to advanced level, were few.

^4^
According to Blum-Kulka *et al.* (1989: 15), social distance is familiarity, which indicates the closeness of the relationship between speaker and hearer. If the relationship is not distant, it could be a family member or friend; if the relationship is distant, it could be someone one does not usually greet, or it could be a stranger.

^5^
Regarding hedged performative, Blum-Kulka *et al.* (1989: 287) stated that the illocutionary verb denoting the requestive intent is modified by modal verbs or verbs expressing intention. Hedged performative in Blum-Kulka *et al.* (1989) is created not by inserting a lexical hedge adverb ( *e.g.*, *somehow, kind of* ) into the explicit performative’s sentence, but by adding an auxiliary verb or a verbal phrase to the explicit performative verb.

^6^
According to Blum-Kulka *et al.* (1989) and Trosborg (1995), hint consists of strong hint and mild hint. Blum-Kulka *et al.* (1989) considered the strong hint to be more direct than the mild hint and placed them in separate categories, while Trosborg (1995) placed them in one category.

^7^
We found one example of an obligation statement in the Uzb. data.

^8^

In this study, only the form yameyoo, a morpheme of desire -yoo is added to the verb yameru “stop,” which functions as a want statement, was observed. That this morpheme implies desire was described in Yamashita (2014: 93, 98).

^9^
As with hedged performative in footnote 5, the sentence hedged by adverbs was not classified as hedged infrigment-indication.

^10^
According to Hashimoto *et al.* (1992: 132), the word *ikenai* is the result of a value judgment about rules. Therefore, this study positioned the “Wrong to Verb”-form as being due to the speaker’s value judgment.

^11^
This category’s examples consist of examples that could be classified not only as apologizing for warning ( *e.g.*, *Mooshiwake arimasen ga* “I’m sorry but,” *Sumimasen ga* “Sorry but,”), but also as both apologizing for warning and alerters ( *e.g.*, *Sumimasen* “Sorry/Excuse me”). One cannot strictly classify the examples of “Sorry” as either an apology for a warning or as an alerter. This study labeled the examples that could be interpreted either way as an apology. Whether an apology for a warning or an alerter, the speakers probably said “Sorry” to convey something that was difficult for them to say.
